# Unconscious improvement in foreign language learning using mismatch negativity neurofeedback: A preliminary study

**DOI:** 10.1371/journal.pone.0178694

**Published:** 2017-06-15

**Authors:** Ming Chang, Hiroyuki Iizuka, Hideki Kashioka, Yasushi Naruse, Masahiro Furukawa, Hideyuki Ando, Taro Maeda

**Affiliations:** 1 Center for Information and Neural Networks (CiNet), National Institute of Information and Communications Technology and Osaka University, Iwaoka, Iwaoka-cho, Nishi-ku, Kobe, Hyougo, Japan; 2 Graduate School of Information Science and Technology, Osaka University, Yamadaoka, Suita, Osaka, Japan; 3 Graduate School of Information Science and Technology, Hokkaido University, Nishi Chome, Kita, Kita-ku, Sapporo, Hokkaido, Japan; Dalhousie University, CANADA

## Abstract

When people learn foreign languages, they find it difficult to perceive speech sounds that are nonexistent in their native language, and extensive training is consequently necessary. Our previous studies have shown that by using neurofeedback based on the mismatch negativity event-related brain potential, participants could unconsciously achieve learning in the auditory discrimination of pure tones that could not be consciously discriminated without the neurofeedback. Here, we examined whether mismatch negativity neurofeedback is effective for helping someone to perceive new speech sounds in foreign language learning. We developed a task for training native Japanese speakers to discriminate between ‘l’ and ‘r’ sounds in English, as they usually cannot discriminate between these two sounds. Without participants attending to auditory stimuli or being aware of the nature of the experiment, neurofeedback training helped them to achieve significant improvement in unconscious auditory discrimination and recognition of the target words ‘light’ and ‘right’. There was also improvement in the recognition of other words containing ‘l’ and ‘r’ (e.g., ‘blight’ and ‘bright’), even though these words had not been presented during training. This method could be used to facilitate foreign language learning and can be extended to other fields of auditory and clinical research and even other senses.

## Introduction

When adults learn a new foreign language, it is difficult for them to perceive differences between speech sounds that are not part of their native language. For example, native Japanese speakers are usually unable to perceive the difference between the “l” and “r” sounds in English [1–5]. Similarly, Mandarin tones are difficult for English speakers to perceive [6]. The ability to distinguish phonetic stimuli and form categories is essential for speech perception. Recognition patterns specific to a language need to be encoded in the memory. These language-specific memory traces and categories develop for native languages during early childhood [7], thus enabling the perception and discrimination of native [8], but not non-native, speech sounds. If two different categories of speech sounds in a foreign language are encompassed by a single native category, it becomes very difficult for an adult studying that language to perceive the difference, and extensive training is required for them to learn this ability.

A recent study [9] presented a potential to develop an unconscious learning. Their study indicated that visual perceptual learning was achieved using decoded fMRI neurofeedback without stimulus presentation. However, this technique requires participants to discriminate the target in advance.

Some studies have shown that auditory discrimination ability can be improved by behavioral training during which mismatch negativity (MMN) becomes stronger and serves as an index of sound-discrimination accuracy [10–12]. The MMN is an event-related potential (ERP) that involves a negative voltage shift of baseline electroencephalographic (EEG) activity at the frontocentral and central scalp electrodes in response to new or novel sounds [13, 14]. The component of the waveform is obtained by subtracting the ERP to the standard stimuli from that to the deviant stimuli in an oddball event. The MMN usually peaks at about 100–250 ms from change onset [15, 16]. The MMN can be elicited by any discriminable auditory change and provides an objective measure of discrimination accuracy for practically any separate dimension of auditory stimulation [8, 16–25]. Interestingly, the MMN response can be detected in the absence of any conscious awareness of a difference [8]. Furthermore, MMN can be elicited without the listener subjectively attending to the sound stimuli [11, 23]. These features of MMN have been combined with neurofeedback in recent studies [26, 27] showing that participants could unconsciously achieve a significant improvement in the auditory discrimination of pure tones that could not be discriminated previously. On the other hand, MMN can be elicited by changes in complex stimuli such as speech sounds [24, 25], so we think that discrimination of speech sounds may be improvement using MMN neurofeedback. Furthermore, the sounds of words have each character, so there is another possibility that recognition ability for individual words also would be an improvement. However, speech sounds are more complex than pure tones. The contrast (e.g., ‘light’ and ‘right’) has different consonants, but also includes the same vowel part that could mask the non-native consonants. Therefore, it is not known whether MMN neurofeedback is effective for speech sound as well as pure tones, or whether this is relevant to learning a foreign language.

Here, we investigated the effectiveness of using MMN neurofeedback for discriminating speech sounds (‘light’ and ‘right’ were the target words for learning) and recognizing individual sounds (‘light’ or ‘right’). Furthermore, we focused on verifying whether the neurofeedback learning could be effective for other words containing “l” and “r” sounds (e.g., ‘blight’ and ‘bright’), even though those words had not been presented during training. We assumed that our neurofeedback method is effective for speech sound as well as pure tones, and relevant to learning a foreign language. That is, after training, participants should be better-able to correctly discriminate and recognize words containing “l” and “r” sounds than control participants who did not receive neurofeedback.

## Methods

The experimental design employed a pretest–posttest procedure closely modeled after the procedure used by previous study [15]. This procedure consists of a pre-test phase, a training phase, and post-test phase. The pre-test was the same as post-test, consisting of a behavioral auditory discrimination (BAD) test and a behavioral auditory recognition (BAR) test. In the training phase, each participant underwent 5 days of training, which were completed over 10 days with at least 24 hours between sessions. BAD and BAR tests for learning sounds (detail in section of stimuli) were always performed once after the end of training on each day. Besides, after the end of the experiment, an oral report of participants was required about the question "how do you made the disc size change?"

### Participants

Fifteen subjects participated in the present study (8 males). All participants were right-handed, monolingual speakers of Japanese (age range, 22–37 years), and all had never lived outside Japan. They began studying English in school at about 12 years of age. Most of their exposure to English had taken place in the classroom. No participants reported a history of hearing or speech disorders.

The participants were randomly distributed into the two groups: the neurofeedback group (5 men, 3 women) and the control group (3 men, 4 women). All participants gave written informed consent, and the study protocol was approved by the local ethics research committee at Osaka University, Japan. Additionally, all research was performed in accordance with the ethical standards described in the Declaration of Helsinki.

### Stimuli

Twenty-two sets of stimulus materials were used in the experiment: (1) Learning sounds: the synthesized sounds “light” and “right” with a duration of 440 ms, including 10 ms rise and fall times, were used for MMN recording in training procedure, pre- and post-test, BAD and BAR test after training in each training day. (2) No-learning sounds: other 21 sets of synthesized sounds of words containing the consonants “l” or “r” were used for only BAR test in pre- and post-test. The intensity of all stimuli was 85 dB. Stimuli were presented binaurally via earphones.

### Behavioral auditory discrimination (BAD) test

**Fig 1:**
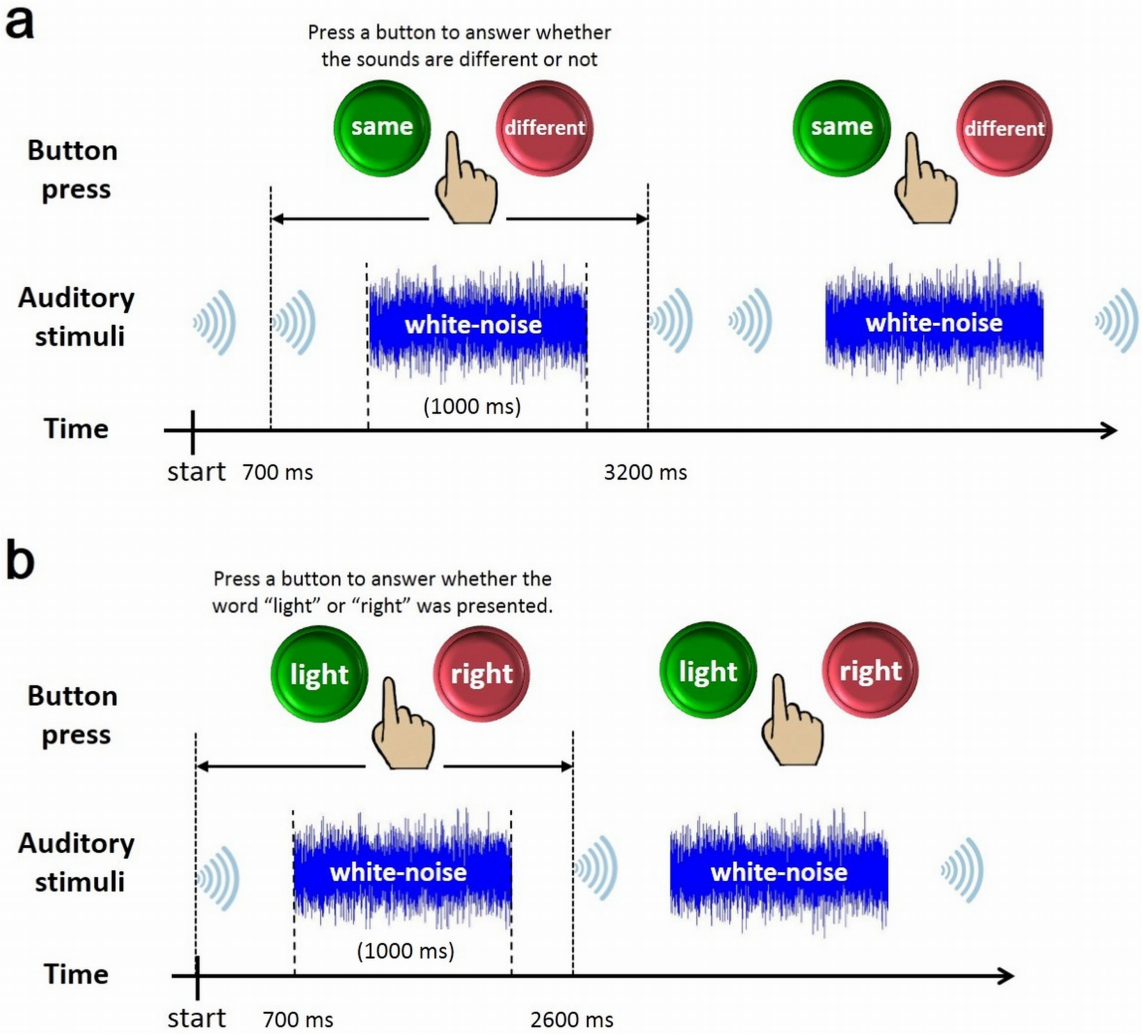
Schematic drawing of behavioral auditory ability tests; (a) Schematic showing two trials in the behavioral auditory discrimination (BAD) test; (b) Schematic showing two trials in the behavioral auditory recognition (BAR) test.

Behavioral auditory discrimination ability was assessed with a two-alternative forced choice task. In the BAD test, two words were presented as a stimulus set in one of four combinations (“light” and “right”; “light” and “light”; “right” and “right”; and “right” and “light”). The SOA in BAD test was the same as those in MMN recording. The order of presentation of the combinations was randomly determined and counterbalanced across trials (the number of trials for each combination was controlled to be equal). Throughout the task, the participants were asked to fixate on a solid green disc with a 0.8 degree of visual angle (hereafter written as degree) radius at the center of the monitor. After each trial, a 2.06 s interval was inserted, consisting of 1 s of white noise as sound interference between 0.53 s silence periods (Fig 1A). During these intervals, the participants reported whether the two words presented in a trial were different by pressing one of two buttons on a keyboard. All participants always pressed the button using their right-hand. Participants were given a brief break after each run of 40 trials. The participants performed 80 trials on each experiment day. No feedback was given to the participants about the results of the test.

### Behavioral auditory recognition (BAR) test

Like the BAD test, behavioral auditory recognition ability was also assessed with a two-alternative forced choice task. In the BAR test, one word was presented as a stimulus. The order of the presentation of words was randomly determined and counterbalanced across trials (the number of trials for each word was controlled to be equal). Throughout the task, the participants were asked to fix their eyes on a solid green disc with a 0.8-degree radius at the center of the monitor. After each trial, a 2.16 s interval was inserted, consisting of 1 s of white noise as sound interference between 0.58 s silence periods (Fig 1B). During these intervals, the participants reported which consonant (“l” or “r”) was in the presented word by pressing one of two buttons on a keyboard. All participants always pressed the button using their right-hand. Participants were given a brief break after each run of 40 trials. The participants performed 80 trials for the target words “light” and “right” on each day and performed 176 trials for all words (target and non-target) on their first and last days. No feedback was given to the participants about the results of the test.

### EEG processing and analysis

**Fig 2:**
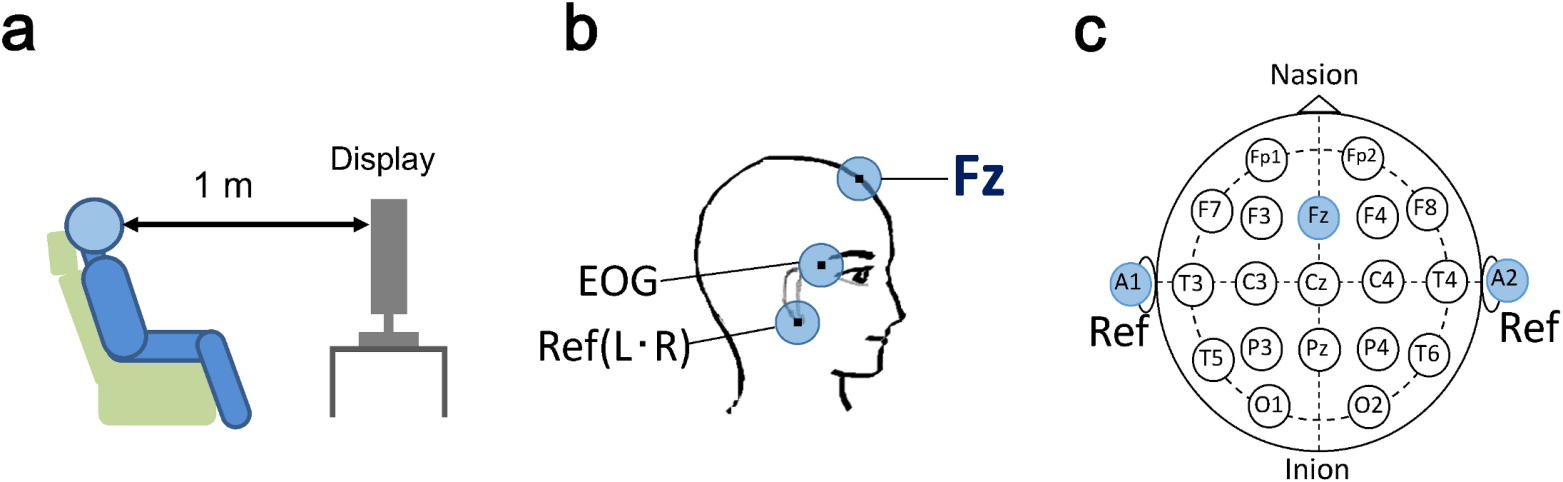
Experiment details; (a) The experimental setup; (b) The locations of EEG and EOG electrodes; (c) The International 10–20-system for EEG electrode placement.

In MMN recording of the learning stage, an auditory stimulus sequence with the words “light” and “right” as the standard and deviant stimuli, respectively, was presented in an oddball paradigm. The stimulus onset asynchrony (SOA)—time between the onsets of stimuli, was the 700 ms. The total number of trials was 300 (“light,” 240 trials; “right,” 60 trials) in each session. The stimuli were presented in a random order. EEG responses were measured with an MP150 Data Acquisition System (BIOPAC Systems Inc., Goleta, CA, USA) and Ag/AgCl pad electrodes. Signals from the electrodes were recorded at a sampling rate of 500 Hz and band-pass filtered online at 0.1–35 Hz. A ground electrode for EEG recordings was placed on the forehead. The reference electrodes were placed on each ear, and the reference was average between electrodes on the two ears. EEG were recorded at the Fz electrode (using the International 10–20-system for EEG electrode placement; Fig 2B and 2C) over 600 ms starting at stimulus onset and including a 100 ms pre-stimulus interval, which served as the baseline. Voltage variations caused by vertical eye movements were monitored with an electrode attached to the upper- outer edge of the left eye. Recordings that contained voltage variations of ±40 μV due to vertical eye movements were omitted. The ERP were averaged across trials separately for each condition, and the MMN was obtained by subtracting the average standard ERP from the average deviant ERP. MMN peak latencies were measured from the most negative peak at Fz at 100–250 ms post-stimulus. MMN amplitudes were calculated as the peak absolute value in the grand-average waveform. However, the calculation was only done for negative values, and the values became zero if the peak was positive.

### Training procedure

For the training sessions in the learning stage, participants were seated in an antistatic chair in front of a 23-inch computer screen (Fig 2A). A program written in visual C++ was used for presenting the visual feedback and auditory stimuli.

**Fig 3:**
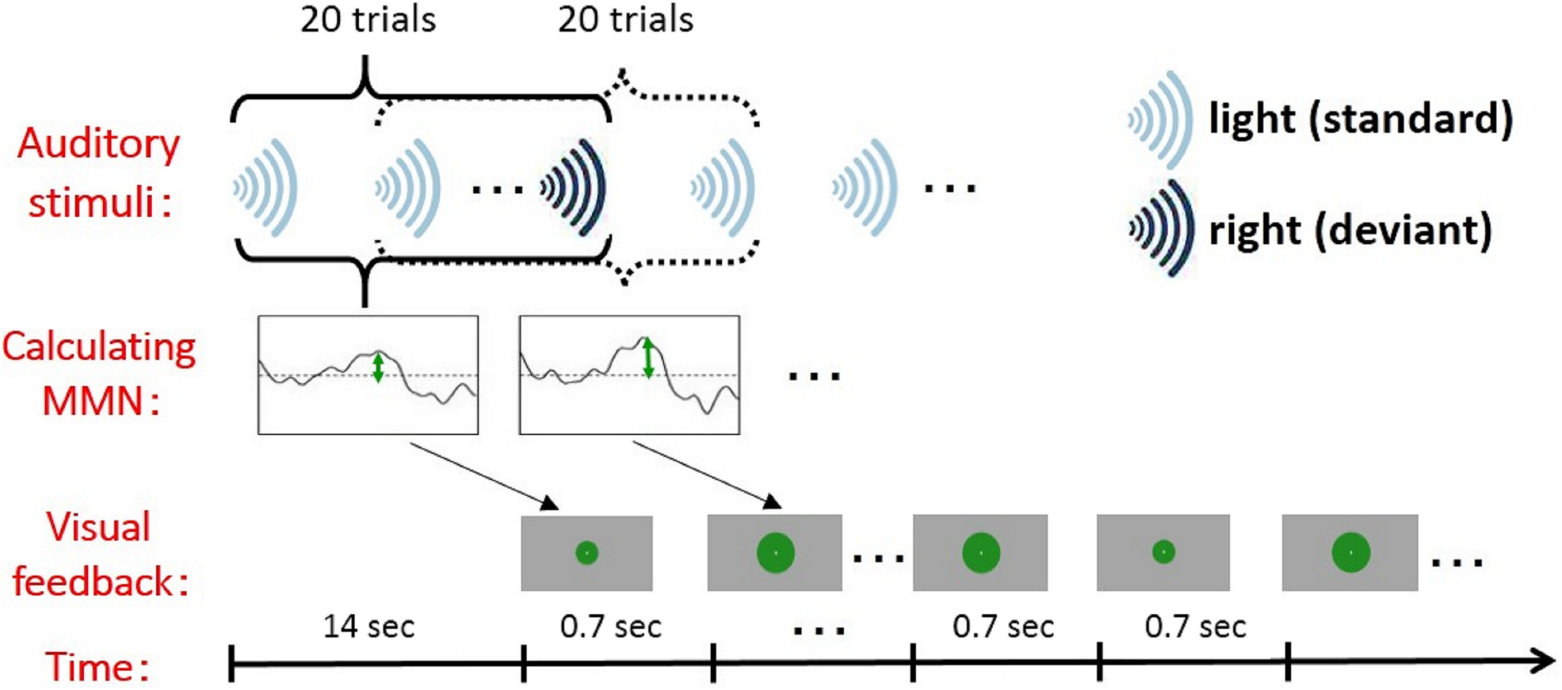
The procedure for neurofeedback training.

In the neurofeedback training experiment, participants were instructed to ignore sounds played through their earphones and concentrate on making the solid green disc as large as possible. The radius of the green disc was fixed for the first 20 stimuli in a session. The average amplitude of the MMN for these trials was calculated (16 standard and 4 deviant stimuli), and beginning with trial 21, the radius of the disc corresponded to the amplitude of this MMN. The MMN was updated every 0.7 s along with the auditory stimuli. The size (here means radius) of the disc (0.4–4.97 degrees radius) was determined every 0.7 s by mapping the MMN amplitude linearly (Fig 3). A single session consisted of a sequence of 210 s (total 300 trials: “light,” 240 trials; “right,” 60 trials), and 12 sessions were conducted on each training day. The participants in the control group were given the same stimuli and instructions, but the sizes of the green discs they were shown did not correspond to their MMN responses. Instead, the sizes corresponded to the sequences of visual stimulus presented to participants in the neurofeedback group. Rather, the participants did not know whether they were in the neurofeedback or control group.

### Preliminary experiment

In order to determine maximum of the feedback disc in training procedure, we have to know the goal (maximum value of the negative amplitude) that we want participants to fulfil, we conducted a preliminary experiment in which the MMN was calculated using an auditory stimulus sequence of 1000 Hz and 2000 Hz tones as the standard and deviant stimuli in an oddball paradigm. These two tones are easily discriminable from each other; therefore, the absolute value of the negative amplitude of the MMN for the two tones was used as the maximum value (preMAX), and the disc’s maximum possible radius was set to 4.97 degrees [14,15]. The minimum size of the feedback disc is the size of the white fixation point (0.4 degrees) presented in the center of the display. The calculation formula was $SIZE=4.57\;*\frac{MMN}{preMAX}+0.4$. The size could not become larger than the maximum possible size even if the MMN was greater than the preMAX, and if the negative peak did not occur, the disc was set to the minimum size.

## Results

### Improvement in the BAD test

**Fig 4:**
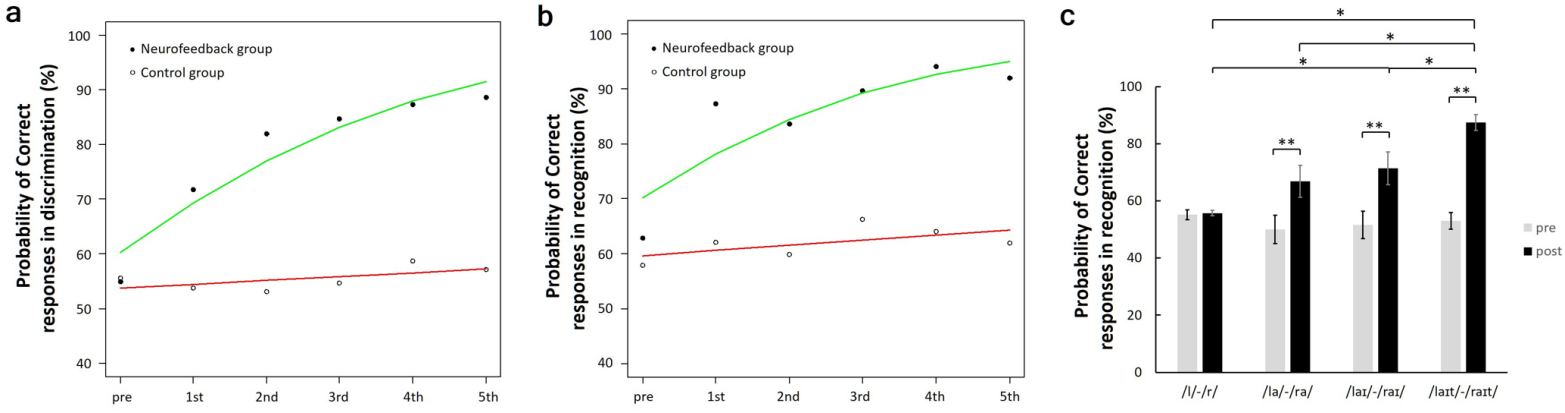
Results of the behavioral auditory ability tests and changes in neural activity; (a) Average probability of correct responses (neurofeedback group: solid circles; control group: empty circles) and logit models (neurofeedback group: green line; control group: red line) in discrimination and (b) Average probability of correct responses (neurofeedback group: solid circles; control group: empty circles) and logit models (neurofeedback group: green line; control group: red line) in recognition for the words “light” and “right”; (d) Average probability of correct responses in recognition for 22 words with the consonants “l” or “r” on pre-test (gray bars) and post-test (black bars) assessments of the neurofeedback group(*n = 8*); The error bars indicate the *SEM*; * *p* < 0.05, ** *p* < 0.01.

To evaluate improvement in participants’ auditory perception, a BAD test was performed before the first day of training (pre-test) and at the end of each training day. In the BAD test, participants were asked whether two words (the same auditory stimuli that were used for training) were different (Fig 1A). Probability of correct responses in the BAD test was compared between the pre- and post-test stages. Because the outcomes of BAD data are dichotomous, we analyzed differences in outcomes after NF training with logistic regression. Fig 4A shows probability of correct responses and learn model that fitting a logistic function for the neurofeedback and control groups. Scores on the pre-test were not significantly different from chance (50% correct), as determined by a binomial test (the critical score for a significant difference was 57.1%). However, outputs indicated that days training is significantly associated with the probability of discriminating the two sounds in the neurofeedback group (*p* < 0.001, OR = 1.48, 95%CI = 1.41–1.56) but not in the control group (*p* = 0.161, OR = 1.03, 95%CI = 0.99–1.07).

### Improvement in the BAR test for learned words

In addition to the BAD test, a BAR test was performed before the first day of training (pre-test) and at the end of each training day. In the BAR test, a single-sound stimulus was presented to participants, who were then asked whether the word “light” or “right” was presented. The presented word was randomly selected from the sound stimuli (“light” or “right”; Fig 1B). The participants’ probability of correct responses in the BAR test was compared in the pre- and post-test stages. we also analyzed differences in outcomes after neurofeedback training with logistic regression. Fig 4B shows probability of correct responses and learn model that fitting a logistic function for the neurofeedback and control groups. And the outputs indicated that days training is significantly associated with the probability of recognizing sound (“light” or “right”) in the neurofeedback group (*p* < 0.001, OR = 1.52, 95%CI = 1.43–1.61) but not in the control group (*p* = 0.056, OR = 1.04, 95%CI = 1–1.08).

### Improvement in the BAR test for non-learned words

In addition to the improvement in behavioral performance for the learned words “light” and “right,” we also assessed whether the there was an improvement in behavioral recognition of other non-learned words containing the consonants “l” or “r.” The BAR test for non-learned words was performed before the first day of training (pre-test) and after the last training day (post-test). The presented word was randomly selected from the sound stimuli in the word list (Table 1), and participants were asked whether the consonant in the presented word was “l” or “r.” Non-learned words were distributed into four classes according to the commonality of a phoneme: /lait/-/rait/ (“light” and “right,” “blight” and “bright”), /lai/-/rai/ (“fly” and “fry”), /la/-/ra/ (“glass” and “grass”), /l/-/r/ (the rest of the words). The probability of correct responses in recognition for non-learned words was also tested. Two-way [class × test stage] repeated measures ANOVA indicated significant main effects of test stage (*F*(1, 92) = 51.74, *p* < 0.01) and class (*F*(3, 92) = 6.54, *p* < 0.01), and a significant interaction between class and test stage (*F*(3, 78) = 7.71, *p* < 0.01). There was no significant difference in recognition performance between pre-and post-tests for the /l/-/r/ class. However, we found a significant improvement in recognition performance between pre-and post-test results for the /la/-/ra/ class (*F*(1, 92) = 11.42, *p* < 0.01), /lai/-/rai/ class (*F*(1, 92) = 15.92, *p* < 0.01), and /lait/-/rait/ class (*F*(1, 92) = 47.50, *p* < 0.01). In addition, Fig 4C shows a significant difference in probability of correct responses in recognition on post-test between classes using Bonferroni's correction for multiple comparisons (*MSe* = 134.9191, *p* < 0.05).

**Table 1:** The list of words used in the BAR test (learned words are in boldface).

Classes	Words
/lait/-/rait/	**light-right**, blight-bright
/lai/-/rai/	fly-fry
/la/-/ra/	glass-grass
/l/-/r/	lamp-ramp, lane-rain, land-rand, late-rate, leach-reach, lead-read, leap-reap, led-red, lest-rest, let-ret, link-rink, load-road, lock-rock, long-wrong, lope-rope, flesh-fresh, blues-bruise, pleasant-present

### Improvement in neural activity

**Fig 5:**
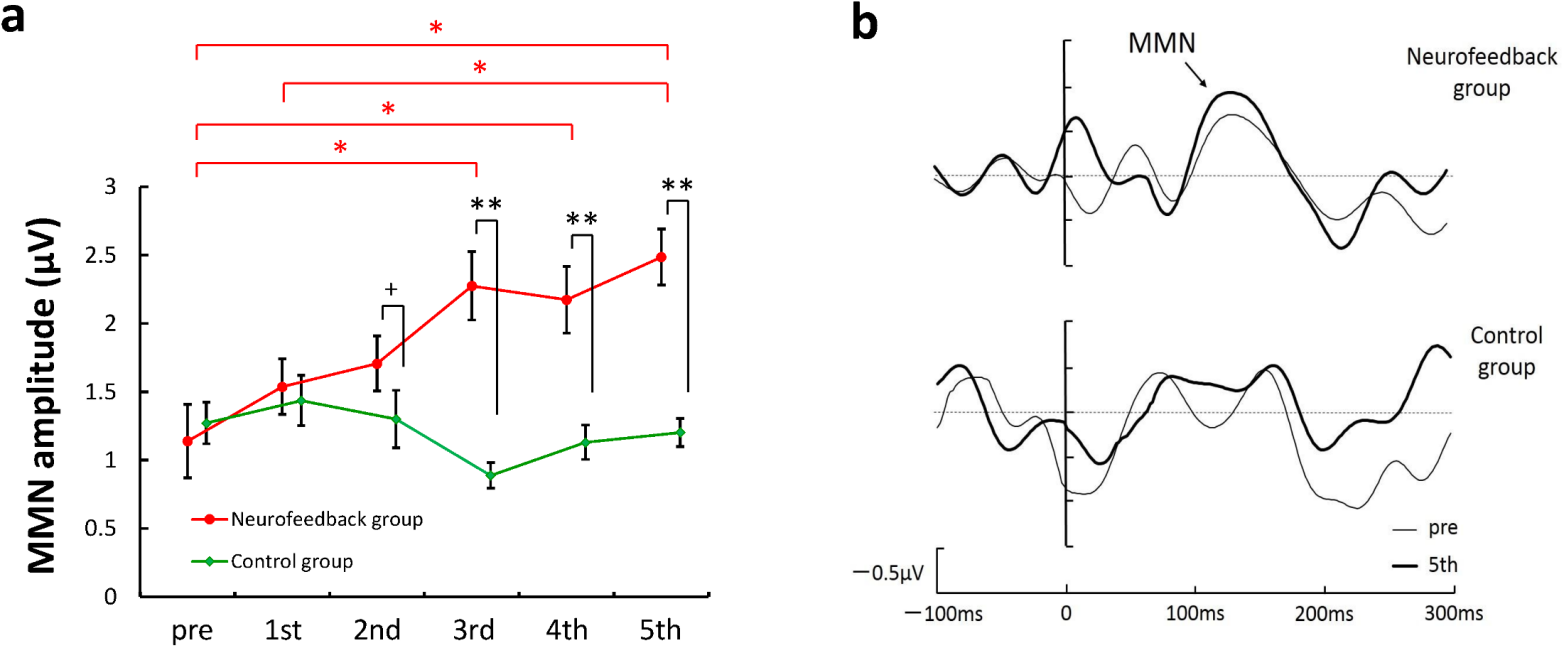
Results of the changes in neural activity; (a) Average MMN amplitudes on the pre-test and on each training day in the neurofeedback group (red circles) and control group (green rhombi); The error bars indicate the *SEM*; * *p* < 0.05, ** *p* < 0.01. (b) The group grand average MMN responses on the pre-training and 5^th^ training days in the neurofeedback and control groups respectively.

In addition to improvements in behavioral performance, we assessed whether neural activity changed in the neurofeedback group. Using electroencephalography (EEG) data collected during the pre-test and on each training day, we calculated and compared the average MMN amplitudes of the neurofeedback and control groups. Two-way [group × training stage] repeated measures ANOVA indicated a significant main effect of group (*F*(1, 78) = 33.13, *p* < 0.01), a marginal main effect of training stage (*F*(5, 78) = 2.12, *p* < 0.1), and a significant interaction between group and training stage (*F*(5, 78) = 4.92, *p* < 0.01). Fig 5A shows that there was no significant difference in MMN amplitude in the pre-training between the neurofeedback and control groups. However, we found a marginal difference in average MMN amplitude on the second training day (*F*(1, 78) = 1.97, *p* < 0.1) and significant differences in average MMN amplitudes on the third, fourth, and fifth days (3rd: *F*(1, 78) = 22.89, *p* < 0.01; 4th: *F*(1, 78) = 12.94, *p* < 0.01; 5th: *F*(1, 78) = 19.59, *p* < 0.01). Fig 5A also shows significant improvements on the third, fourth, and fifth training days compared with the pre-test using Bonferroni's correction for multiple comparisons (*MSe* = 0.3141, *p* < 0.05). Fig 5B shows the group grand average MMN responses on the 1st and 5th training days in the both groups, respectively. However, due to the difference in peak latency between participants, the result shown in Fig 5B differs somewhat from Fig 5A.

After the experiment was completed on the last training day, participants were asked how they made the disc size change, and none of their responses was related to the speech sounds in the experiment. For example, they gave explanations such as “I tried to imagine the solid green disc coming closer to my eyes” and “I tried to imagine putting air into the green balloon.”

## Discussion

In this study, we examined whether MMN neurofeedback is effective for speech sound as well as pure tones, or whether this is relevant to learning a foreign language. We did a training task for helping native Japanese speakers learn to distinguish between ‘light’ and ‘right’ in English. First of all, our results indicate that participants can learn to discriminate the speech sounds ‘light’ and ‘right’ without any explicit training. Secondly, it is possible for adults to learn to recognize new sounds that do not exist in their native language. Thirdly, another result of this study is that neurofeedback learning is effective for words containing the consonants “l” and “r” besides the learned words of ‘light’ and ‘right’, although some of them had no significant learning effect. The result proposed a possibility that discrimination ability can be extended to novel words. Above all, MMN neurofeedback is useful for adults to learn foreign languages.

A previous study in perceptual learning reported that repetitive pairing of reward and visual stimuli leads to performance improvements for those stimuli [28]. In our training experiment, participants were instructed to concentrate on making the solid green disc as large as possible, and the size of solid green disc changed every 0.7 s after the first 20 trials. Therefore, there is a possibility that the simple visual stimuli (size of the disc) had worked as a reinforcement signal leading to behavioral improvements. Furthermore, although the participants were asked to ignore the auditory stimuli during training, we hypothesized that they might become accustomed to hearing the stimuli repeatedly, and, thus, learning might unconsciously occur and auditory discrimination performance might improve. However, the results of behavioral auditory test and neural activity in the control group contradict this idea. The visual and auditory stimuli during training were the same between groups, but the sizes of the green discs they were shown correspond to participants' MMN responses in the neurofeedback group but not in the control group. And no improvements in behavioral performance or neural activity were found in the control group. This result shows that improvements in behavioral performance and neural activity in the neurofeedback group were caused by the neurofeedback rather than simply by the repeated stimuli those did not correspond to neural activity (MMN responses).

Previous studies have shown that in some behavioral auditory-discrimination training tasks, the MMN has increased following behavioral training [11,29–32]. Our recent findings [26, 27] have indicated that auditory-discrimination performance for pure tones can be improved by enhancing brain activity without behavioral discrimination training. The results of the present study further indicate that an improvement in the discrimination of complex sounds at the word level was elicited by the same training framework [26,27]. Furthermore, besides discrimination ability, recognition ability was improved through the neurofeedback training. Before training, the participants could not discriminate between the words “light” and “right.” Because the two categories are encompassed by a single native category (“light”), they perceive the two words as the same. It is thought that language learners can perceive a new category after learning to perceive the differences between two categories. Despite the fact that the participants were not given any feedback after the BAD and BAR tests, none of them performed at probability of correct responses under 50% in the BAR test. This means that nobody mistook the two words for each other. It is possible that this is because the participants had experience studying English in school starting at 12 years of age, and they might have known the characteristics of the sound differences as prior knowledge.

In this study, we also assessed improvements in the recognition of other words with the consonants “l” or “r,” even though they had not been presented during training. Our results show that probability of correct responses in recognition for words was improved along with an increase in recognition of the shared phoneme. The transfer for the training was better for words in which the same vowel “ai” followed r/l. The result suggests that training improved pairing consonant-vowel discrimination, rather than just consonants. A previous study [12] examined backward masking effects on non-native consonants by a following vowel using magnetoencephalography to measure mismatch negativity in response to synthesized speech sounds. The sound pressure lever of vowels is higher than that of consonants so that vowels including the transitional part from a consonant to a vowel possibly mask the consonant. Their results indicated that the backward masking effect on non-native consonants by following vowels may be one reason for the difficulties in learning foreign consonants, such as /r/ for Japanese. Therefore, it is conceivable that discrimination between the words in the /lait/-/rait/, /lai/-/rai/, and /la/-/ra/ classes was improved because the backward masking effect for these words was suppressed in our training. Conversely, the backward masking effect on the words in the /l/-/r/ class, which do not necessarily have a vowel in common, could not be completely suppressed using neurofeedback training. As a solution, we propose that simply more training is required for recognizing these words.

A previous study has shown that behavioral training improves the probability of correct responses in recognition for /l/ and /r/ [33]. Participants responded by pressing “1” or “2” to identify the spoken word as containing “r” or “l,” and feedback was provided in the form of different signals. Nevertheless, it is important to note that probability of correct recognition responses only improved by 16 percentage points on average, which is still substantially poorer than the near-perfect probability of correct identification responses achieved by native English speakers. Furthermore, the training phase took place over a period of 3–4 weeks. Similar training procedure and result were observed in another study of Japanese adults learning English [34]. In the study [34], participants responded by pressing response buttons marked "S" and "D." Immediate feedback was given by lights that were illuminated over the correct response button. In the both aforementioned study, a feedback paradigm was used in which a binary (correct/incorrect) assessment of behavioral responses was provided to participants in a standard form of behavioral training. Our results in the present study, however, indicate that auditory-discrimination performance for words can be improved by enhancing brain activity without behavioral discrimination training. The probability of correct identification responses for the target words “light” and “right” improved by an average of 35% following a 5-day training period. In particular, probability of correct recognition responses also improved by an average of 34%. When we view the learning curves in more detail, we find that the probability of correct discrimination responses improved by over 30 percentage points on average within the first three days of training. The probability of correct responses in discrimination and recognition, MMN amplitudes were all significantly improved on the third training day compared with the pre-test, and did not change significantly thereafter. There are quite a few possible reasons why behavioral discrimination training often becomes difficult. For example, the feedback in a standard behavioral training paradigm is limited to a binary (correct/incorrect) assessment of behavioral responses. However, supraliminal behavioral responses are not necessarily identical with the subliminal brain responses occurring when participants are not conscious of differences. For example, regardless of how the brain processes the sounds, the feedback received is “incorrect” when the behavior is wrong. Such information cannot be used to accurately evaluate the learning process, as it obscures how learning occurs. By contrast, our neurofeedback method provides continuous feedback demonstrating the accuracy of the current state and guiding learners to improve their brain processes and ultimately their discrimination ability. Interestingly, as in our previous research [26,27], the participants in our study were not aware of the purpose of the experiment. Because this new neurofeedback method does not require learners to pay attention to the auditory stimuli [35,36] or to be aware of the learning process, it can unconsciously improve discrimination ability in foreign-language learning.

## Conclusion

Our results indicate that adults can learn to discriminate the speech sounds of foreign languages unconsciously without any behavioral training. In addition, it is possible for adults to learn to recognize individual sounds that do not exist in their native language. The neurofeedback method was also effecting for some words containing the consonants “l” and “r” besides the learned words. Therefore, we think that our neurofeedback method has a promising nature that may supersede previous behavioral training for foreign language learning. Furthermore, our method also has the potential to be developed into an unconscious-learning device in the form of a brain-computer interface (BCI) game that people can simply enjoy using for learning target sounds in a foreign language with a specific goal that can be achieved by a large MMN while unconsciously improving their listening ability. However, this method can only be useful for limited auditory training at present. Our future work will focus on the learning effect of our method to other auditory fields (such as learning by a musician or sound engineer), or clinical therapies (such as for hearing impairments or schizophrenia). Moreover, the basic concepts underlying our findings could potentially be extended to other senses if the biomedical signal used for detecting the secondary clue can be acquired for neurofeedback.

## References

[1] Kuhl PK. Learning and representation in speech and language. Curr Opin Neurobiol. 1994; 4: 812–822. 7888763 10.1016/0959-4388(94)90128-7

[2] Yamada RA, Tohkura YI. The effects of experimental variables on the perception of American English /r/ and /l/ by Japanese listeners. Percept Psychophys. 1992; 52: 376–392. 1437471 10.3758/bf03206698

[3] Logan JS, Lively SE, Pisoni DB. Training Japanese listeners to identify English /r/ and /l/: A first report. J Acoust Soc Am. 1991; 89: 874–886. 2016438 10.1121/1.1894649 PMC3518834

[4] Lively SE, Pisoni DB, Yamada RA, Tohkura YI, Yamada T. Training Japanese listeners to identify English /r/ and /l/. III. Long-term retention of new phonetic categories. J Acoust Soc Am. 1994; 96: 2076–2087. 7963022 10.1121/1.410149 PMC3518835

[5] Phillips C, Marantz A, McGinnis M, Pesetsky D, Wexler K, Yellin E. et al Brain mechanisms of speech perception: A preliminary report. MIT Working Papers in Linguistics. 1995; 26: 125–163.

[6] Wang Y, Spence MM, Jongman A, Sereno JA. Training American listeners to perceive Mandarin tones. J Acoust Soc Am 1999; 106: 3649–3058. 10615703 10.1121/1.428217

[7] Cheour M, Ceponiene R, Lehtokoski A, Luuk A, Allik J, Alho K, et al Development of language-specific phoneme representations in the infant brain. Nature Neurosci. 1998; 1: 351–353. 10196522 10.1038/1561

[8] Näätänen R, Lehtokoski A, Lennes M, Cheour M, Huotilainen M, Iivonen A, et al Language-specific phoneme representations revealed by electric and magnetic brain responses. Nature. 1997; 385: 432–434. doi: 10.1038/385432a0 9009189 10.1038/385432a0

[9] Shibata K., Watanabe T., Sasaki Y. &Kawato M. Perceptual learning incepted by decoded fMRI neurofeedback without stimulus presentation. Science. 2011; 334: 1413–1415. doi: 10.1126/science.1212003 22158821 10.1126/science.1212003 PMC3297423

[10] Näätänen R, Paavilainen P, Tiitinen H, Jiang D, Alho K. Attention and mismatch negativity. Psychophysiology. 1993; 30: 436–450. 8416070 10.1111/j.1469-8986.1993.tb02067.x

[11] Tremblay K, Kraus N, McGee T. Neurophysiological changes during speech-sound training. NeuroReport. 1998; 9: 3557–3560. 9858359 10.1097/00001756-199811160-00003

[12] Koyama S, Gunji A, Yabe H, Yamada RA, Oiwa S, Kubo R, et al The masking effect in foreign speech sounds perception revealed by neuromagnetic responses. Neuroreport. 2000; 11: 3765–3769. 11117487 10.1097/00001756-200011270-00034

[13] Näätänen R, Gaillard A.W, Mäntysalo S. Early selective-attention effect on evoked potential reinterpreted. Acta Psychol. 1978; 42: 313–329. 10.1016/0001-6918(78)90006-9 685709

[14] Näätänen R, Paavilainen P, Rinne T, Alho K. The mismatch negativity (MMN) in basic research of central auditory processing: a review. Clin Neurophysiol. 2007; 118: 2544–2590. doi: 10.1016/j.clinph.2007.04.026 17931964 10.1016/j.clinph.2007.04.026

[15] Amenedo E, Escera C. The accuracy of sound duration representation in the human brain determines the accuracy of behavioural perception. Eur J Neurosci. 2000; 12: 2570–2574. 10947831 10.1046/j.1460-9568.2000.00114.x

[16] Sams M, Paavilainen P, Alho K, Näätänen R. Auditory frequency discrimination and event-related potentials. Electroencephalogr Clin Neurophysiol /Evoked Potentials Section. 1985; 62: 437–448. 10.1016/0168-5597(85)90054-1 2415340

[17] Tiitinen H, May P, Reinikainen K, Näätänen R. Attentive novelty detection in humans is governed by pre-attentive sensory memory. Nature. 1994; 327: 90–92. 10.1038/372090a0 7969425

[18] Rüsseler J, Altenmüller E, Nager W, Kohlmetz C, Münte T. F. Event-related brain potentials to sound omissions differ in musicians and non-musicians. Neurosci Lett. 2001; 308: 33–36. 11445279 10.1016/s0304-3940(01)01977-2

[19] Tervaniemi M, Saarinen J, Paavilainen P, Danilova N, Näätänen R. Temporal integration of auditory information in sensory memory as reflected by the mismatch negativity. Biol Psychol. 1994; 38: 157–167. 7873700 10.1016/0301-0511(94)90036-1

[20] Tervaniemi M, Winkler I. Pre-attentive categorization of sounds by timbre as revealed by event‐related potentials. NeuroReport. 1997; 8: 2571–2574. 9261829 10.1097/00001756-199707280-00030

[21] Sonnadara R. R, Alain C, Trainor L. J. Occasional changes in sound location enhance middle latency evoked responses. Brain Res. 2006; 1076: 187–192. doi: 10.1016/j.brainres.2005.12.093 16487494 10.1016/j.brainres.2005.12.093

[22] Bertoli S, Smurzynski J, Probst R. Temporal resolution in young and elderly subjects as measured by mismatch negativity and a psychoacoustic gap detection task. Clin Neurophysiol. 2002; 113: 396–406. 11897540 10.1016/s1388-2457(02)00013-5

[23] Alho K, Woods DL, Algazi A. Processing of auditory stimuli during auditory and visual attention as revealed by event-related potentials. Psychophysiology. 1994; 31: 469–479. 7972601 10.1111/j.1469-8986.1994.tb01050.x

[24] Pettigrew C.M, Murdoch B.E, Ponton C.W, Finnigan S, Alku P, Kei J. et al Automatic auditory processing of English words as indexed by the mismatch negativity, using a multiple deviant paradigm. Ear Hear. 2004; 25: 284–301. 15179119 10.1097/01.aud.0000130800.88987.03

[25] Sams M, Aulanko R, Aaltonen O, Näätänen R. Event-related potentials to infrequent changes in synthesized phonetic stimuli. J Cogn Neurosci. 1990; 2: 344–357. doi: 10.1162/jocn.1990.2.4.344 23964759 10.1162/jocn.1990.2.4.344

[26] Chang, M, Iizuka, H, Naruse, Y, Ando, H, Maeda, T. An interface for unconscious learning using mismatch negativity neurofeedback. In: AH. '14 Proceedings of the 5th Augmented Human International Conference; 2014 March; New York, NY, USA. New York: ACM; 2014.

[27] Chang M, Iizuka H, Naruse Y, Ando H, & Maeda T. Unconscious learning of auditory discrimination using mismatch negativity (MMN) neurofeedback. Sci Rep. 2014; 4: 6729 doi: 10.1038/srep06729 25342520 10.1038/srep06729 PMC4208041

[28] Seitz AR, Kim D, Watanabe T. Rewards evoke learning of unconsciously processed visual stimuli in adult humans. Neuron. 2009; 61: 700–707. doi: 10.1016/j.neuron.2009.01.016 19285467 10.1016/j.neuron.2009.01.016 PMC2683263

[29] Kraus N, McGee T, Carrell TD, King C, Tremblay K, Nicol T. Central auditory system plasticity associated with speech discrimination training. J. Cognitive Neurosci. 1995; 7: 25–32. 10.1162/jocn.1995.7.1.25 23961751

[30] Näätänen R, Schröger E, Karakas S, Tervaniemi M, Paavilainen P. Development of a memory trace for a complex sound in the human brain. NeuroReport. 1993; 4: 503–506. 8513127 10.1097/00001756-199305000-00010

[31] Winkler I, Kujala T, Tiitinen H, Sivonen P, Alku P, Lehtokoski A, et al Brain responses reveal the learning of foreign language phonemes. Psychophysiology. 1999; 36: 638–642. 10442032

[32] Pantev C, Roberts LE, Schulz M, Engelien A, Ross B. Timbre-specific enhancement of auditory cortical representations in musicians. Neuroreport. 2001 12; 169–174. 11201080 10.1097/00001756-200101220-00041

[33] Bradlow AR, Pisoni DB, Akahane-Yamada R, Tohkura YI. Training Japanese listeners to identify English/r/and/l: IV. Some effects of perceptual learning on speech production. J Acoust Soc Am. 1997; 101: 2299–2310. 9104031 10.1121/1.418276 PMC3507383

[34] Strange W, Dittmann S. Effects of discrimination training on the perception of/r-l/by Japanese adults learning English. Percept Psychophys. 1984; 36: 131–145. 6514522 10.3758/bf03202673

[35] Näätänen R. The mismatch negativity: a powerful tool for cognitive neuroscience. Ear Hear. 1995; 16: 6–18. 7774770

[36] Sussman E, Winkler I, Wang W. MMN and attention: competition for deviance detection. Psychophysiology. 2003; 40: 430–435. 12946116 10.1111/1469-8986.00045

